# Filarial Excretory-Secretory Products Induce Human Monocytes to Produce Lymphangiogenic Mediators

**DOI:** 10.1371/journal.pntd.0002893

**Published:** 2014-07-10

**Authors:** Tiffany Weinkopff, Charles Mackenzie, Rob Eversole, Patrick J. Lammie

**Affiliations:** 1 Division of Parasitic Diseases, Centers for Disease Control and Prevention, Atlanta, Georgia, United States of America; 2 Department of Cell Biology, University of Georgia, Athens, Georgia, United States of America; 3 Department of Pathobiology and Diagnostic Investigation, Michigan State University, East Lansing, Michigan, United States of America; 4 Department of Biological Sciences, Western Michigan University, Kalamazoo, Michigan, United States of America; University Clinic Bonn, Germany

## Abstract

The nematodes *Wuchereria bancrofti* and *Brugia* spp. infect over 120 million people worldwide, causing lymphedema, elephantiasis and hydrocele, collectively known as lymphatic filariasis. Most infected individuals appear to be asymptomatic, but many exhibit sub-clinical manifestations including the lymphangiectasia that likely contributes to the development of lymphedema and elephantiasis. As adult worm excretory-secretory products (ES) do not directly activate lymphatic endothelial cells (LEC), we investigated the role of monocyte/macrophage-derived soluble factors in the development of filarial lymphatic pathology. We analyzed the production of IL-8, IL-6 and VEGF-A by peripheral blood mononuclear cells (PBMC) from naïve donors following stimulation with filarial ES products. ES-stimulated PBMCs produced significantly more IL-8, IL-6 and VEGF-A compared to cells cultured in medium alone; CD14^+^ monocytes appear to be the primary producers of IL-8 and VEGF-A, but not IL-6. Furthermore, IL-8, IL-6 and VEGF-A induced *in vitro* tubule formation in LEC Matrigel cultures. Matrigel plugs supplemented with IL-8, IL-6, VEGF-A, or with supernatants from ES-stimulated PBMCs and implanted *in vivo* stimulated lymphangiogenesis. Collectively, these data support the hypothesis that monocytes/macrophages exposed to filarial ES products may modulate lymphatic function through the secretion of soluble factors that stimulate the vessel growth associated with the pathogenesis of filarial disease.

## Author Summary

Lymphatic filariasis is caused by parasitic worms with approximately 120 million people infected worldwide and over 1 billion people at risk. The adult worms reside in host lymphatic vessels (LV) but most infected individuals do not present with overt clinical symptoms. Individuals exhibiting lymphedema, a common form of the disease, are often antigen negative; however, infected individuals, though often asymptomatic, have dilated LVs suggesting that early damage to the lymphatic architecture may lead to lymphedema in these infected individuals. In the LVs, adult worms release excretory-secretory (ES) products. Filarial ES products do not directly activate lymphatic endothelial cells (LEC), so we hypothesized that accessory cells may activate LECs indirectly and contribute to the development of disease. Here, we show that adult filarial ES products induce human blood cells, specifically monocytes, to produce lymphangiogenic factors such as IL-8 and VEGF-A and that these factors induce the formation of LVs *in vivo*. These results support a role for filarial ES products in altering the lymphatic architecture in filarial-infected individuals and this may contribute to LV pathology and the development of lymphedema.

## Introduction

Lymphatic vessels (LVs) are important components of a system vital to the body's maintenance that includes immune surveillance and fat absorption; the primary function of these vessels is to drain excess interstitial fluids and to prevent tissue swelling [1]. Lymphangiectasia is a condition in which LVs are abnormally dilated and this pathology is often associated with the development of lymphedema, when lymphatic fluid becomes stagnant and leaks back into the surrounding interstitium. Lymphatic dilation may result from a variety of causes including trauma, cancer-related treatment regimes such as lymphadenectomy, and genetic mutations in FOXC2 or VEGFR-3. However, the majority of lymphatic pathology seen worldwide is associated with the filarial nematode parasites, *Wuchereria bancrofti* and *Brugia malayi* which cause lymphedema in millions of individuals.

An estimated 120 million people worldwide are infected by filarial parasites [2]. Lymphatic filariasis is an infection with varying degrees of clinical disease, where infected individuals can exhibit overt clinical symptoms such as lymphedema and hydrocele or be asymptomatic yet with microfilaremia. Although these asymptomatic microfilaremic individuals do not display any overt clinical manifestations, they do present with hidden sub-clinical complications [2], [3] such as dilated and tortuous lymphatics [4], [5], and scrotal lymphangiectasia in men [6], [7]. Ultrasonographic examination of the scrotal region of 14 asymptomatic Brazilians revealed that 50% of microfilaremic individuals demonstrated lymphatic dilation and tortuosity [8]. In microfilaremic individuals, abnormal lymphatics are present in 69% of limbs by static lymphoscintigraphy and in 100% of limbs by dynamic flow lymphoscintigraphy, which are sensitive indicators of lymphatic dysfunction [4], [5], [9]. In addition, studies of superficial skin punch biopsies have revealed that 78% and 68% of limbs from patients with clinical disease and asymptomatic microfilaremia, respectively, contained LVs that were abnormally dilated [5], [10]. More recently, it was also demonstrated that children as young as three years of age can present with lymphangiectasia as measured by lymphoscintigraphy suggesting that sub-clinical pathology can occur at a very early age [11].

The causes for the lymphatic dilation in filarial-infected individuals remain unknown, but lymphangiectasia is seen in SCID mice infected with *Brugia* suggesting that the worm and/or innate mechanisms, and not the host's adaptive immune system, are involved in the development of lymphatic dilation [12], [13]. Furthermore, the dilation can be reversed in nude mice by removing or killing the adult worms [14], [15]. An important finding was made by Shenoy et al. who showed that there is a reduction in lymphatic dilation following worm death induced by DEC treatment [11], [16].

The molecules involved in the proliferation and maintenance of endothelial cells (EC) are a family of growth factors known as the vascular endothelial growth factors (VEGF) as well as cytokines such as IL-3, IL-6, IL-7 and IL-8 [17]–[25]. VEGF-A, VEGF-C and VEGF-D, and their corresponding receptors, have all been shown to support lymphatic endothelial cell (LEC) proliferation, migration, survival and tubule formation; thus these molecules are potent regulators of lymphangiogenesis [17], [26]. Several studies have shown that plasma levels of these lymphangiogenic factors, including VEGF-A, VEGF-C, VEGF-D and angiopoietins, are significantly elevated in filarial-infected individuals including those with filarial lymphedema compared to endemic normal control subjects [27], [28]. Elevated plasma levels of VEGF-A were also seen in individuals with hydrocele [29]. Furthermore, human infection with the filarid, *Onchocerca volvulus*, induces lymphangiogenesis in parasite-containing nodules [30] and this neovascularization is associated with the expression of lymphangiogenic molecules such as VEGF-C [31].

Monocytes/macrophages appear to be the predominant producers of the VEGFs and the presence of monocytes/macrophages has been correlated with lymphangiogenesis [32]–[36]. In human onchocercal nodules, some mononuclear cells expressed both the macrophage marker, MAC-1, and the lymphatic-specific marker, LYVE-1, and these double-positive cells were integrated into the lymphatic endothelium [30], [31]. Thus in this present study we have addressed the role of monocytes/macrophages contributing to the production of lymphangiogenic mediators in response to filarial ES and their influence on lymphatic ECs.

## Materials and Methods

### Ethical Statement

For human studies, informed consent was obtained from all human subjects and approved by the Institutional Review Board at CDC. For animal studies August rats, imported from the MRC London, UK and bred locally at Western Michigan University, and maintained in standard animal laboratory housing conditions, were used. All animals were anesthetized using isoflurane gaseous equipment (Summit Medical Equipment Company, Foster City, CA). The animals were housed individually for the course of the experiment in the Animal Facilities of Western Michigan University. All the animal procedures were approved by the Western Michigan University Animal Use and Care Committee (IACUC) under project 10-01-07 before the project was begun. The study conformed to the Guide for the Care and Use of Research Animals published by the National Research Council.

### Parasite ES Products

*Brugia malayi* adult female worms were collected from the peritoneal cavity of infected jirds, *Meriones unguiculatus*, obtained from the NIAID Filariasis Research Reagent Repository at the University of Georgia (Athens, GA). Worms were isolated 4–12 months post infection from jirds and some of the adult females will have been gravid at this point. For the collection of ES products, 50 live adult female worms were cultured *in vitro* for 7 days at 37°C in 10 mL serum-free RPMI 1640 media (GIBCO) supplemented with 2 mM L-glutamine and antibiotics (100 U/mL penicillin and 100 µg/mL streptomycin). Supernatants were collected and fresh medium added daily. Supernatants containing the ES products were centrifuged at 1000× g for 10 min to remove the microfilariae and the microfilariae were resuspended in PBS and counted to ensure worm viability. Supernatants were then concentrated with a Centricon filter (Millipore, Bedford, MA) to a volume of ∼300 µL. This process resulted in ∼670 ng/mL of worm protein. ES products were stored at 4°C until further use. Male worms were not used because they do not secrete the same quantity of protein material as females (unpublished observations). Prior to cell stimulations with ES products, ES products were filtered using 0.45 µm Millex-HA syringe filters (Millipore, Carrigtwohill, Ireland) and used in a dose-dependent (diluted at 1∶10, 1∶50) manner across various replicates and batches. A batch is defined as a specimen containing the concentrated ES products from 50 female worms over one week pooled together. All batches of ES products were tested for endotoxin activity using the Limulus Amebocyte Lysate QCL-1000 assay (Lonza, Walkersville, MD) and ES products were only used for experiments when endotoxin concentrations were ≤0.1 EU/mL.

### Isolation of Peripheral Blood Mononuclear Cells

Human PBMCs were isolated using lymphocyte separation media (MP Biomedicals, Solon, OH) as directed by the manufacturer. In brief, blood was collected from normal healthy donors by venipuncture in 10 mL EDTA Vacutainer tubes (Becton Dickinson, Franklin Lakes, NJ). After centrifugation the buffy coat was removed, resuspended in RPMI 1640 media supplemented with 10% FBS (Atlas Biologicals, Fort Collins, CO), 2 mM L-glutamine and antibiotics and layered over lymphocyte separation media. Cells were centrifuged for 30 min at 1000× g at 4°C, the buffy coat was removed, washed and cells were counted using a hemocytometer.

Human CD14^+^ monocytes were enriched by positive selection from PBMCs using CD14^+^ MACS technology (Miltenyi Biotec, Auburn, CA) as directed by manufacturer. CD14^+^ monocyte isolation was confirmed by flow cytometry using mouse anti-human CD14^+^ PE (BD Pharmingen, San Jose, CA) and CD14^+^ cells were routinely enriched to a purity of 94–98%.

### Culture of LECs

Human dermal lymphatic microvascular endothelial cells (HMVEC-dLy) were purchased from Clonetics (Lonza) and maintained in EBM-2 basal media supplemented with EGM-2 MV SingleQuots (Lonza) according to manufacturer's instructions. Cells were used from passages 4-8.

### Production of Lymphangiogenic Factors by PBMCs and CD14^+^ Monocytes

Cells were plated at 1×10^6^ PBMCs or 5×10^5^ CD14^+^ cells in 500 µL RPMI 1640 media supplemented with 10% FBS, 2 mM L-glutamine and antibiotics and stimulated with or without 100 ng/mL LPS or ES diluted at 1∶10 and 1∶50 for 72 h. The final concentration of the ES in the dilutions used to stimulate the human cells was approximately 10 to 67 ng/mL. Cell culture supernatants were collected and analyzed for IL-3, IL-6, IL-7, IL-8 and VEGF-A using the Bio-Plex Pro multiplex suspension array system (Bio-Rad, Hercules, CA) according to the manufacturer's instructions. Data was obtained using low PMT voltage settings and analyzed by the Bio-Plex Manager software version 4.1.1 and concentrations were calculated based on a standard curve derived from a recombinant cytokine standard. If the cytokine level in the sample was higher than the highest value on the standard curve, which occurred in many of the LPS stimulations, the highest value of the standard curve was reported for that data point. All samples were stimulated in parallel with ES products diluted at 1∶10 and 1∶50, but only results from the ES concentration which generated optimal stimulation were reported. VEGF-C and VEGF-D production were analyzed by the Quantikine Immunoassay kits (R&D, Minneapolis, MN) as directed by the manufacturer.

### *In Vitro* Matrigel Tubule Formation by LEC

LECs were released from the flask by gentle trypsinization (Lonza), washed, counted and 1×10^5^ LECs were stimulated in 200 µL EGM-2 MV SingleQuot media devoid of VEGF and spiked with 10 ng/mL IL-6 (R&D), 10 ng/mL IL-8 (Sigma, St. Louis, MO) or 1 ng/mL VEGF-A (R&D) for 10 min at 37°C before seeding. Cells were plated onto 100 µL Growth Factor-reduced Matrigel Matrix (BD Biosciences, Bedford, MA) coating a 24 well plate using the thin gel method as per manufacturer's instructions. After 24 h, 5 randomized fields per well were photographed at 5× magnification on a Zeiss AxioVert 200M microscope (Carl Zeiss, Thornwood, NY). The images were opened and analyzed in AxioVision release 4.7.2. At a scaling ratio of 1∶1 image analysis was performed; the total number of tubules was counted and the length of each tubule measured.

### *In Vivo* Matrigel Tubule Formation Assay

These experiments were carried out in August rats as previous work using this strain of rat demonstrated that the most appropriate time to sample for dermal vascular growth is 9 days [37]. Rat carrier-free recombinant proteins including IL-8, IL-6, VEGF164 were purchased from R&D Systems. Growth Factor-reduced Matrigel was injected into rats with or without 10 ng/mL IL-8, 10 ng/mL IL-6 or 10 ng/mL VEGF-A as directed by the manufacturer. For the injections of the recombinant proteins, 3024 µL of liquid Matrigel was mixed with 576 µL of the recombinant rat lymphangiogenic proteins yielding a final concentration of 10 ng/mL for each protein with each animal receiving a 0.5 mL injection. In addition, we collected supernatants from PBMCs stimulated with or without worm ES products (1∶10) as previously mentioned. Supernatants from 5 different individuals were pooled and 576 µL of the pooled supernatants was mixed with 3024 µL of liquid Matrigel and 0.5 mL of this mix was injected into each rat [38]. These supernatants were analyzed by luminex bead technology using the Bio-Plex 8-plex kit (IL-2, IL-4, IL-6, IL-8, IL-10, GM-CSF, IFNγ, TNFα) as well as IL-5, IL-13 and VEGF (Bio-Rad) according to the manufacturer's instructions. Regardless of Matrigel dilution with either recombinant proteins or PBMC supernatants, the Matrigel concentration was kept constant across all parameters and animals at 6.64 mg/mL.

Six rats were used per group and the various Matrigel-test agent samples were placed in the sub-dermal tissue of the flank using an 18G needle; the same position was used on each anesthetized animal, with only one plug being injected in a single rat. All injections were made by the one person and care taken with each injection to maintain a constant injection pressure and to produce a uniformly distributed plug of the material in the tissues. All rats were observed at least twice daily during the course of the experiment; they tolerated the procedures without any difficulty and did not interfere in any way with the sites where the Matrigel plugs were located.

### Preparation of Histopathology Samples

Animals were sacrificed at day 9 as described above. During plug excision, the skin and underlying tissues/body wall were carefully dissected to observe the status of the plug and the surrounding tissues noting color, presence of scar tissue, vasculature and presence of any abnormal host tissue reaction.

The plugs and adjacent dermal tissues were removed intact from the animals and cut three times through their longest axis to provide three relatively equal slices before fixing. The plugs from each animal were photographed in situ and after removal and sectioning. Tissue sections were then taken from the cut faces of these portions to provide three different areas of each plug for histological preparation and for quantitative assessment. All tissues were fixed in 3.7% buffered formalin for a maximum of 24 h whilst maintaining that the fixation solution remained clear. Tissues were stored for processing in 60% ethanol. Specimens were then processed, embedded in paraffin, and sectioned on a rotary microtome at 4–6 µm. Sections were placed on slides coated with 2% 3-amino-propyl-tri-ethoxysilane and dried at 56°C overnight. Following de-paraffinizing in xylene and hydrating through descending concentrations of ethyl alcohol to distilled water (DW), the slides were placed in Tris-buffered saline (TBS) pH 7.5 (Scytek Labs, Logan, UT) for 5 min for pH adjustment.

### Immunohistochemistry

Following TBS, the podoplanin and IgG test slides underwent heat-induced epitope retrieval utilizing citrate buffer pH 6.0 (Scytek) in a vegetable steamer for 30 min at 100°C, allowed to cool on the counter at RT for 10 min and rinsed in several changes of DW. Von Willebrand Factor VIII (vWF) slides underwent enzyme-induced epitope retrieval utilizing 0.03% Pronase E in TBS for 10 min at 37°C followed by running tap and DW rinses. Prior to test antibody (Ab) use, the sections were subjected to an endogenous peroxidase blocking step (3% hydrogen peroxide/methanol bath for 30 min followed by running tap water and DW rinses), a nonspecific protein blocking step for 30 min (normal horse serum) (Vector Labs, Burlingame, CA), and finally an avidin/biotin blocking system (avidin, Vector Labs; biotin, Sigma Chemicals, St. Louis, MO) for 15 min. Following pretreatment, avidin-biotin complex staining steps were performed at RT on the Dako Autostainer (Dako North America, Inc., Carpinteria, CA). All staining steps were followed by two rinses in TBS+Tween 20 (Scytek). After the sections were rinsed in TBS/Tween20, they were incubated at various times (usually 40–60 min) and various concentrations (from 1 in 40 to 1 in 200) with the various test (primary) Abs. The optimal procedures for each test Ab were determined following assessment under the microscope. Abs were diluted with Normal Antibody Diluent (NAD) (Scytek, Logan, UT). Primary Ab slides were incubated for 60 min with the monoclonal mouse anti-rat podoplanin (ReliaTech/Angio-Proteomie, Boston, MA) diluted 1∶400, or the biotin-conjugated polyclonal rabbit anti-rat IgG (Novus Biologicals, Littleton, CO) diluted 1∶100 in NAD. Slides were rinsed in 2 changes of TBS/Tween20, and then incubated in appropriate biotinylated secondary Ab for the host species of the primary Ab (biotinylated anti-rat, anti-goat, and anti-mouse from Vector Labs) at 10–11.0 µg/mL in NAD incubated for 30 min. The slides were then again rinsed in TBS/Tween20, and then R.T.U. Vectastain Elite ABC Reagent (Vector Labs) was applied for 30 min. The slides were rinsed with TBS/Tween20 and developed using NovaRED peroxidase substrate kit (Vector Labs) for 15 min. After a rinsing in DW, they were finally counterstained using Gill 2 (Lerner) hematoxylin (Thermo Fisher, Kalamazoo, MI), differentiated in 1% aqueous glacial acetic acid, and rinsed in tap water. Slides were then dehydrated, cleared with xylene, and mounted using Flotex permanent mounting media (Thermo Shandon, Pittsburg, PA).

During the establishment of optimal immunostaining for Matrigel sections we compared three antibodies as markers of lymphatic endothelia: a monoclonal mouse anti-rat podoplanin (ReliaTech/Angio-Proteomie, Boston, MA), a monoclonal mouse anti-human D2-40 (DakoCytomation) and a rabbit polyclonal anti-LYVE-1 Ab (Angiobio, Del Mar, CA). From this pre-study we selected the first of these reagents as being the most suitable for quantification. Control tissues used in these studies included rat lymph nodes and dermal neoplasia, and the staining controls included omitting the primary antibody. Routine hematoxylin and eosin staining (H&E) was also employed to examine the tissues and establish the most suitable area for quantification.

### Quantitative Assessment of the Cellular Components of Matrigel Plugs

To avoid any complication from the natural host cellular response to Matrigel, the areas used for cellular assessment for vascular invasion were those central areas free of any overt host cellular response to Matrigel itself; from an examination of all the samples this free area was seen to cover a minimum of 4.0 mm^2^ centered around the mid point of the plug. This central area of the Matrigel plug was quantitatively assessed for the presence of anti-podoplanin and anti-vWF positivity in serial sections. Photographs were taken using bright field and Differential Interference Contrast Microscopy (DIC).

The Chalkley Point Array random sampling technique (The Graticules Ltd. Chalkley Point Array - Model NG52) was used to quantify the immuno-positive staining elements present in the Matrigel plug and thus a relationship to the proportion of the two vascular components present in different groups; the number of points lying over a positively stained entity is statistically proportional to the area occupied by that component [39]. Three areas in each section taken from the three slices of each individual plug were quantified and the number of immuno-positive components was recorded. This provided nine counts for each sample, and thus 54 counts for each treatment group.

In addition, confirmatory values were obtained using a commercial image analysis system - Image-Pro (MediaCybernetics, Bethesda, MD) and Image J (NIH - rsbweb.nih.gov/ij/) and examining the central 4.0 mm^2^ test area of each Matrigel slice. The immunohistochemical staining intensity was standardized for each section by setting the positivity limit for each marker using the respective cell or tissue component in the dermal tissue surrounding the plug in each section. Each area was measured using a pixel color gate (i.e. for marker positive cells), and subtracting the background using collagen tissue as the negative. Triplicate runs were made with the pixel number calculations for each assessment area.

### Statistics

The Signed Rank Test was used in the Statistical Analysis Software (SAS) version 9.1 to compare median cytokine and growth factor production by PBMCs in stimulated and control supernatants. The Signed Rank Test was also used to compare the production of these factors by CD14^+^ monocytes compared to non-CD14 cells. GraphPad Prism 5 software (San Diego, CA) was used to carry out additional statistical analyses to compare the number of tubules per microscopic field in response to stimuli. It was determined from a standard power calculation, a minimum number of animals in each test group to obtain an acceptable significant result was 5; therefore we used 6 animals in each group. The animals were injected in random order and the tissues were assessed blinded to minimize bias. Student's t test and ANOVA were used to assess the results.

## Results

### Filarial ES Products Induce the Production of Lymphangiogenic Factors in PBMCs

**Figure 1:**
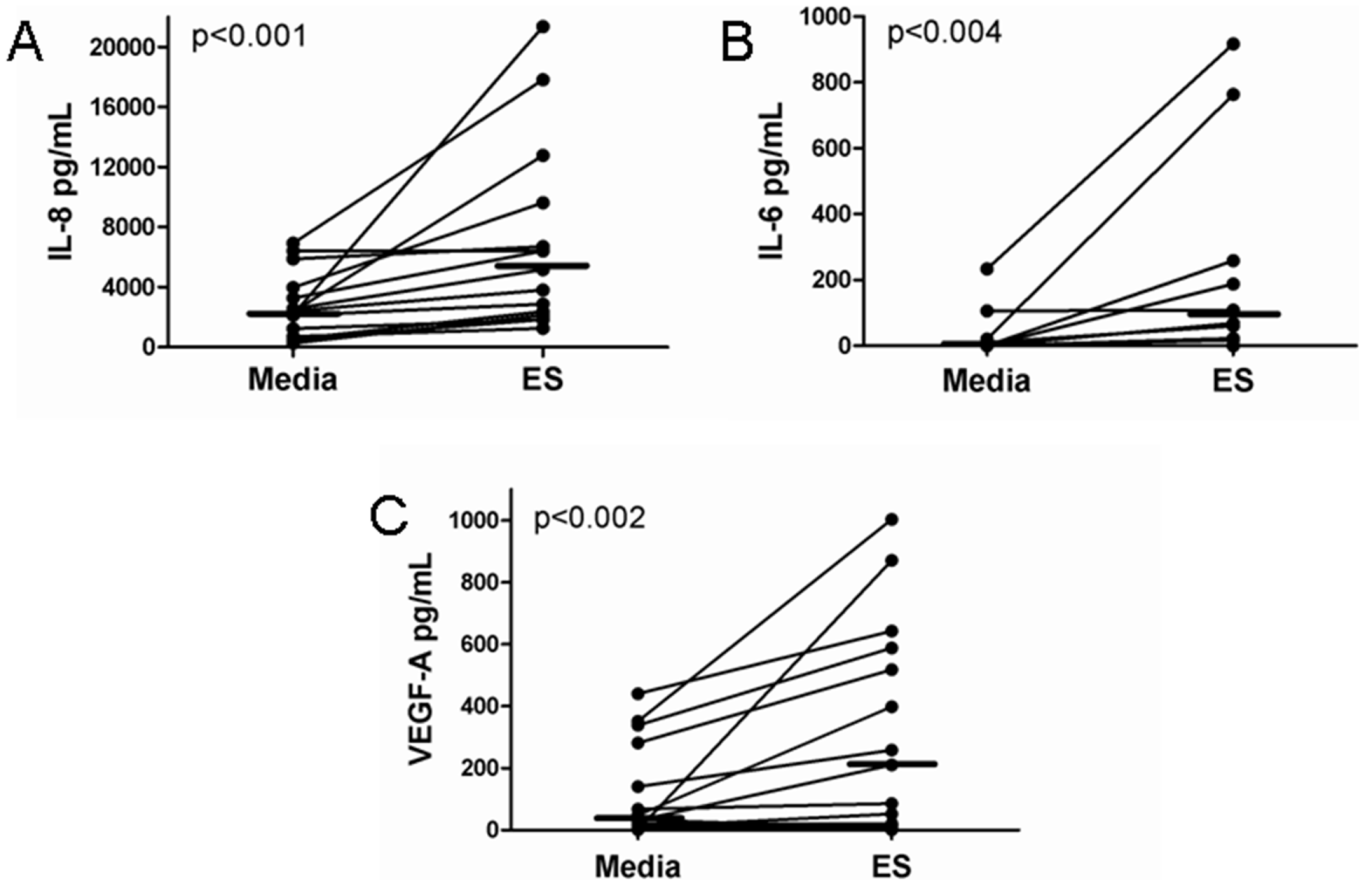
*Brugia* ES products induce the production of lymphangiogenic molecules by human PBMCs. PBMCs were isolated from a minimum of 10 healthy human volunteers and 1×10^6^ cells were stimulated with or without ES for 72 h. Cell supernatants were assessed for the presence of IL-8, IL-6 and VEGF-A by luminex bead analysis. *Brugia* ES products induced the production of (A) IL-8 (n = 15), (B) IL-6 (n = 10) and (C) VEGF-A (n = 15) by PBMCs compared to cells in media alone as assessed by the Signed Rank test. Medians are presented as bars.

We evaluated the ability of *Brugia* ES products to induce the secretion of molecules known to exhibit lymphangiogenic potential in other *in vivo* and *in vitro* settings. Human PBMCs were isolated from healthy volunteers and cultured with or without filarial ES products for 72 h. The supernatant fluids were collected and analyzed for the production of the potentially lymphangiogenic molecules IL-3, IL-6, IL-7, IL-8 and VEGF-A by luminex technology. PBMCs cultured in media alone supplemented with 10% FBS served as a negative control. Cells cultured with filarial ES products secreted significantly higher levels of IL-8, IL-6 and VEGF-A compared to cells cultured in media alone (Fig. 1). We did not detect IL-3 or IL-7 in any of our supernatants. We also attempted to measure VEGF-C and VEGF-D by ELISA, but these were below the limit of detection. Taken together, these data suggest that *Brugia* ES products are capable of inducing the secretion of lymphangiogenic molecules by circulating PBMCs.

### CD14^+^ Monocytes Are the Primary Producers of the Lymphangiogenic Molecules

**Figure 2:**
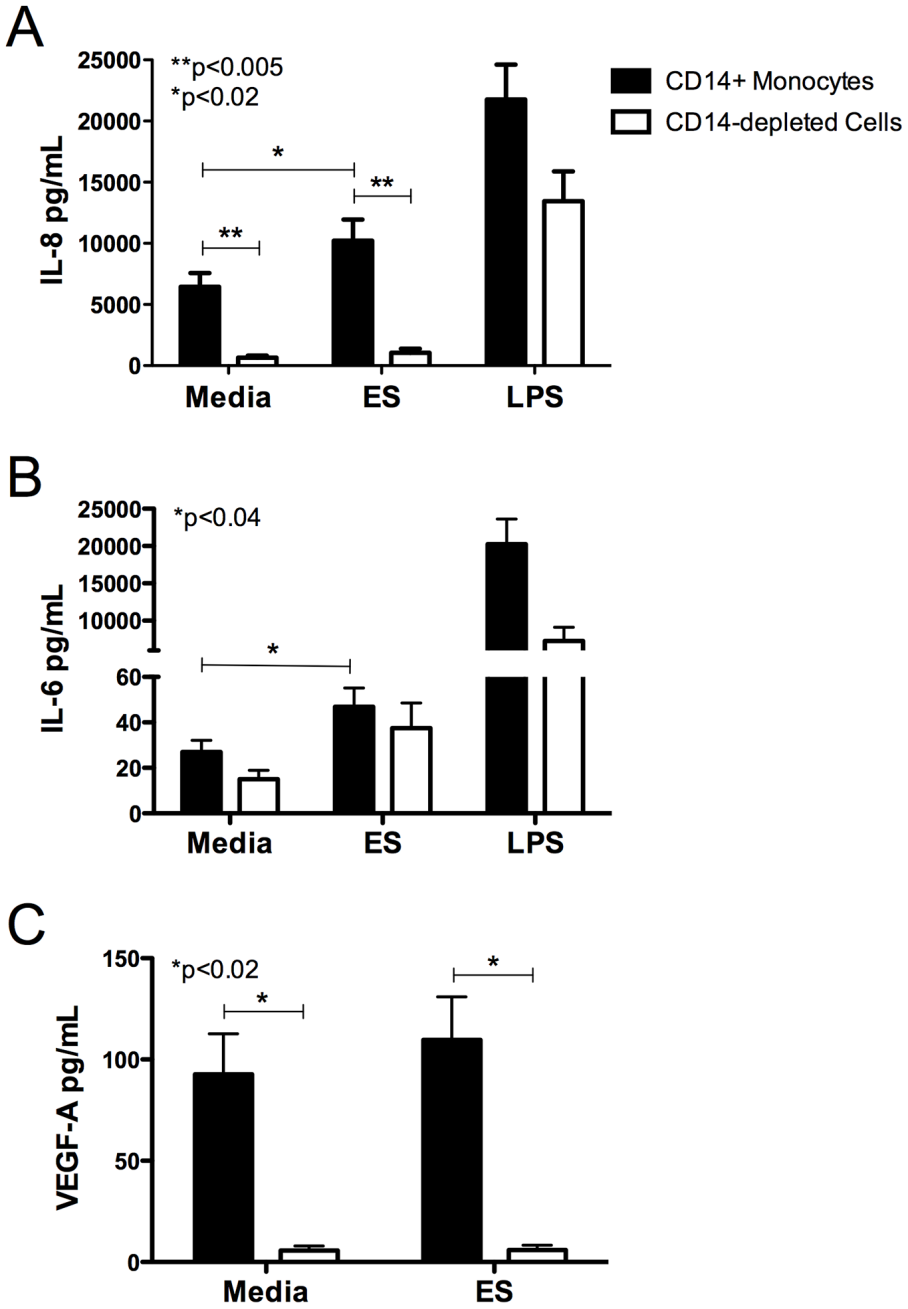
*Brugia* ES products induce the production of IL-8 and VEGF-A by human CD14^+^ monocytes. Human CD14^+^ monocytes were isolated and compared to CD14-depleted cells for IL-8, IL-6 and VEGF-A production in response to worm ES products or LPS. Cell supernatants were assessed for the presence of (A) IL-8 (n = 12), (B) IL-6 (n = 7) and (C) VEGF-A (n = 7) after 72 h of stimulation. Data presented represents the mean +SEM of at least 7 people per factor and comparisons were made using the Signed Rank test. LPS was used as a positive control and stimulated the production of IL-8 (p<0.003) and IL-6 (p<0.02) compared to cells cultured in media alone.

Monocytes/macrophages have been shown to play an important role in the production of VEGFs in tumors and inflammation, so we hypothesized monocytes could be the PBMC in the periphery contributing to the production of IL-8, IL-6 and VEGF-A seen in response to worm ES products. We carried out CD14 fractionation experiments using MACS technology to isolate CD14^+^ monocytes from total PBMCs. As seen in Fig. 2A and 2C, CD14^+^ monocytes secreted significantly higher amounts of IL-8 and VEGF-A compared to CD14-depleted cells in response to filarial ES products. However, CD14-enriched and depleted cell populations produced similar levels of IL-6 (Fig. 2B). CD14^+^ monocytes produced significantly more IL-8 and VEGF-A spontaneously compared to CD14-depleted cells (Fig. 2A and 2C). CD14^+^ monocytes stimulated with *Brugia* ES products also secreted significantly higher levels of IL-8 and IL-6 compared to CD14^+^ cells cultured in media alone. LPS was used as a positive control for the production of IL-8 and IL-6 and robust IL-8 and IL-6 responses were seen following LPS stimulation. Taken together, these data suggest that CD14^+^ monocytes are the primary producers of the lymphangiogenic molecules IL-8 and VEGF-A in response to worm ES products, but CD14^+^ monocytes are not the major cell type contributing to the production of IL-6 in response to worm ES products.

### ES-Induced Lymphangiogenic Mediators Stimulate LECs to Form Tubules *In Vitro*

**Figure 3:**
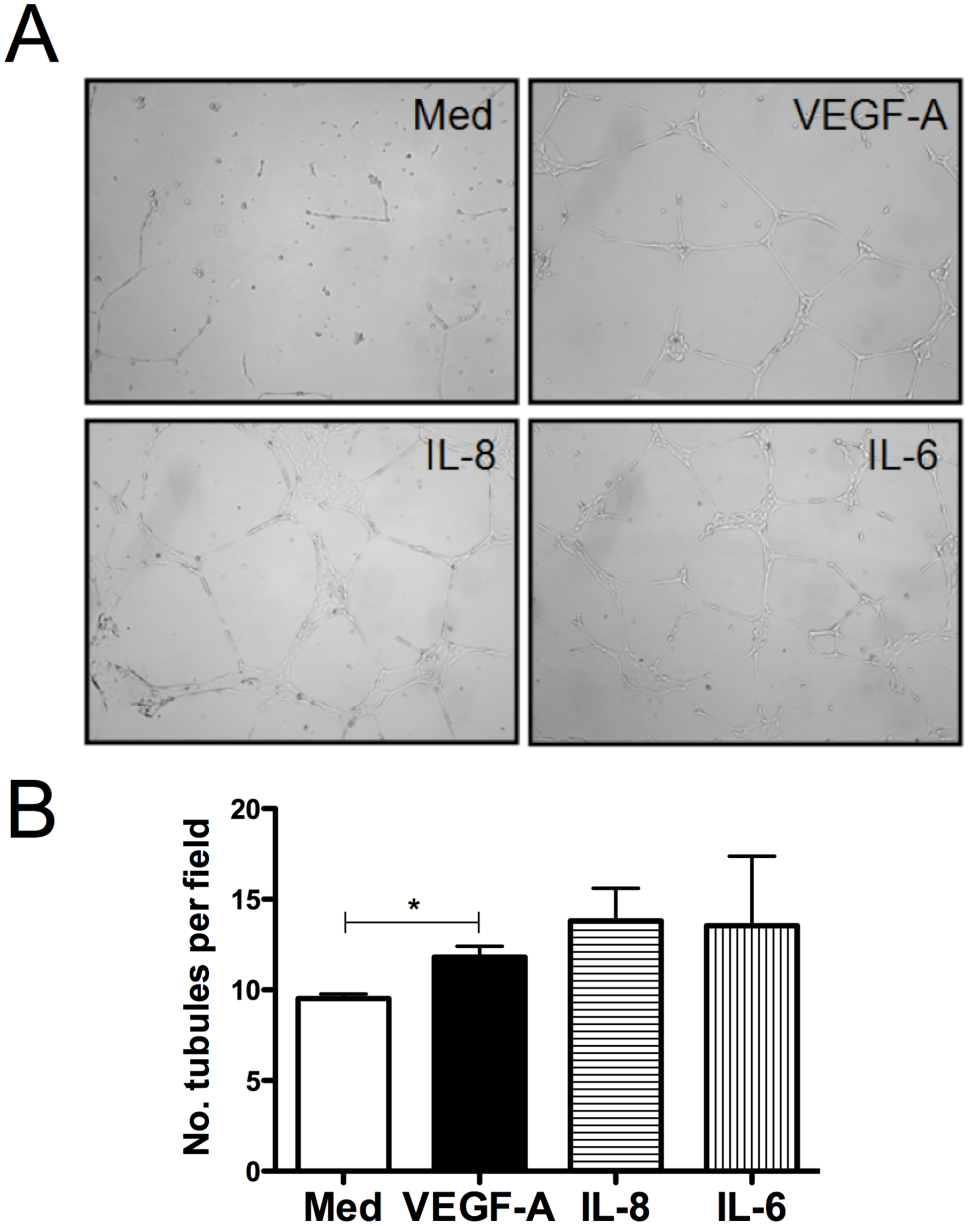
Filarial ES-induced lymphangiogenic mediators induce LEC tubule formation *in vitro*. LECs were grown on Matrigel in the presence or absence of IL-8, IL-6 or VEGF-A and lymphatic networks were photographed (A). (B) The number of tubules was quantified using image analysis software. The data represented here are the means +SEM of one experiment representative of 4 independent experiments performed in triplicate.

Since we were able to demonstrate the production of lymphangiogenic molecules by PBMCs in response to *Brugia* ES products, we examined the ability of these mediators detected following ES stimulation to alter LEC function as measured by tubule formation. LECs were layered on Matrigel cultures and stimulated with concentrations of IL-8, IL-6 and VEGF-A comparable to the amounts detected in supernatants of ES-stimulated PBMCs. After 24 h, LECs cultured in the presence of IL-8, IL-6 and VEGF-A formed a more elaborate tubule network compared to cells cultured in media alone (Fig. 3A). Using image analysis software used to quantify tubule formation, cells cultured in the presence of IL-8, IL-6 or VEGF-A formed a greater number of tubules per microscopic field compared to LECs cultured without stimulus (Fig. 3B).

### ES-Induced Lymphangiogenic Molecules Result in Vascularization of Matrigel Plugs *In Vivo*

Given that mediators produced by PBMCs in response to filarial ES stimulation such as IL-8, IL-6 and VEGF-A induced LEC tubule formation *in vitro*, we hypothesized these molecules could also promote LV formation *in vivo*. To determine if the soluble mediators present in ES-induced supernatants could induce vessel formation *in vivo*, we injected rats with Matrigel containing supernatants from PBMCs (collected from 5 different individuals) that were stimulated with ES products or cultured in media alone. Characterization of the pooled PBMC supernatants which included measurable concentrations of IL-2, IL-6, IL-8 and VEGF is seen in Table 1. In parallel rats were injected with Matrigel containing rat recombinant IL-8, IL-6 or VEGF-A in case the human mediators released by PBMCs in response to filarial ES did not induce a cross species effect and stimulate vessel formation in rats. Given that Matrigel contains a variety of basement membrane proteins including laminin and collagen, Matrigel alone was used as a non-specific protein negative control.

**Table 1:** Cytokine and growth factor levels (pg/mL) in PBMC supernatants^a^.

Group	Unstimulated supernatants	ES-stimulated supernatants^a^
IL-2	3.07	4.41
IL-4	Undetectable	Undetectable
IL-5	Undetectable	Undetectable
IL-6	18.58	68.8
IL-8	5061.76	30898.94
IL-10	Undetectable	Undetectable
IL-13	Undetectable	Undetectable
GM-CSF	Undetectable	Undetectable
IFNγ	Undetectable	Undetectable
TNFα	Undetectable	Undetectable
VEGF	46.04	115.65

a 1×10^6^ PBMCs were stimulated with worm ES or cultured in media alone for 72 h.

Supernatants from 5 different individuals were pooled and cytokines and growth factors were analyzed by luminex bead technology. Matrigel plugs were supplemented with 80 µL of the pooled supernatants and used for rat *in vivo* vessel formation experiments.

**Figure 4:**
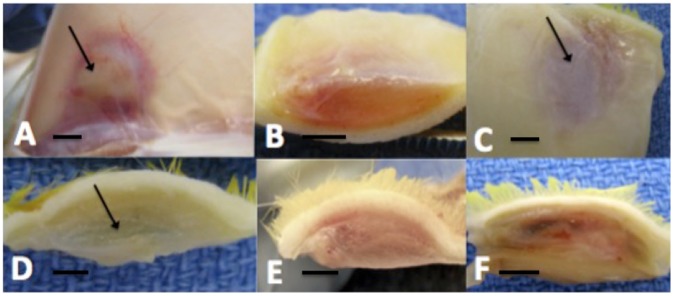
Matrigel plugs in situ. Matrigel was injected into rats with or without 10/mL IL-8, 10 ng/mL IL-6 or 10 ng/mL VEGF-A in a total volume of 0.5 mL. Matrigel was supplemented with supernatants from PBMCs stimulated with filarial ES for 72(A) A subcutaneous Matrigel plug (arrow) containing VEGF-A showing a red-colored vascular response in the surrounding tissues and infiltrating the plug. (B) A cross section of a plug containing filarial ES-PBMC products showing discoloration. (C) Distinct outline of the injected plug (arrow) in the sub-cutaneous tissues. (D) A control Matrigel plug free of coloration. (E and F) Matrigel plugs from IL-6-treated (E) and VEGF-A-treated (F) animals showing a significant vascular response with a dark red area. The scale bars represent 1 cm.

After 9 days, the plugs were excised and subjected to gross inspection for vessel infiltration (Fig. 4A and 4B). Surprisingly, even upon initial gross examination in situ, the Matrigel plugs displayed an overt difference between treated groups and controls. Animals given ES-stimulated PBMC supernatants had increased redness in the plug denoting blood vessel infiltration compared to supernatants from unstimulated PBMCs. Furthermore, rats injected with lymphangiogenic cytokines also had an increased redness compared to Matrigel alone control plugs. The plugs in situ were generally uniform in size and shape. All except one had formed a distinct flattened oval shaped plug; one of six samples from experimental Group 2 was not clearly a round elliptical entity and was dispersed over a wide and indistinct area in the dermis; this was discarded. There was quite considerable variation in color, ranging from yellow-brown to deep pink/red. The control animals showed the yellow-brown end of the spectrum while those in groups receiving lymphangiogenic factors were generally a deeper red color (Fig. 4).

**Figure 5:**
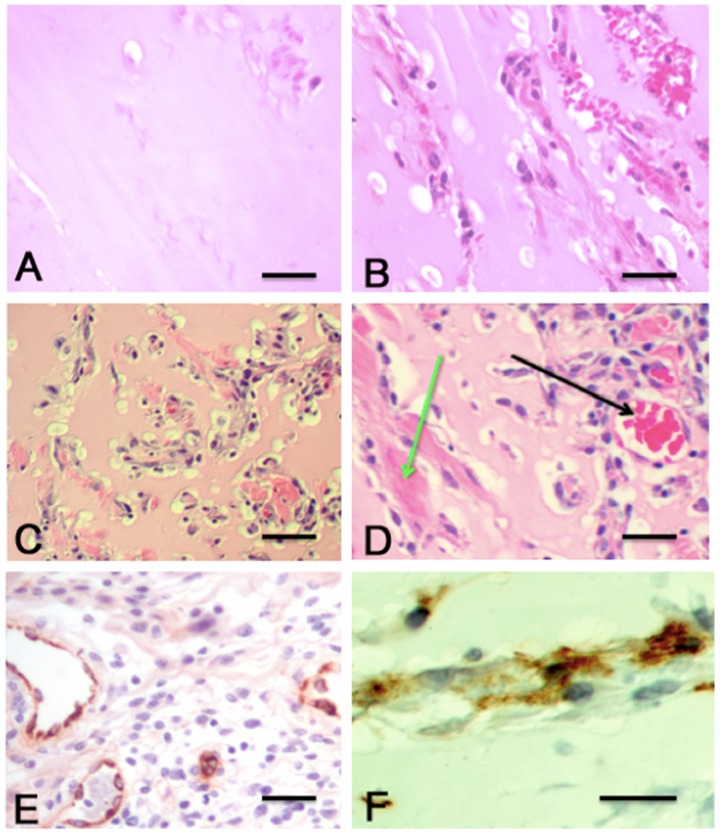
Cellular responses in the central assessment area of Matrigel plugs. Matrigel was injected in the presence or absence of 10/mL IL-8, IL-6 or VEGF-A in 0.5 mL. Matrigel alone was injected as a control. Matrigel was supplemented with supernatants collected from ES-stimulated PBMCs or PBMCs cultured in media as a control and injected into rats. After 9 days, Matrigel plugs were excised, sectioned and analyzed. Representative observations are presented from a single experiment using 6 rats per group. (A) Matrigel alone (control) - H&E stain. (B) Vascular response in VEGF-A plug - H&E stain. (C) Cellular response in PBMC+ES Matrigel plug – H&E stain. (D) Lymphatic vessels (green arrow) together with blood vessels (black arrow) in an IL-6-treated Matrigel plug. (E) Example of anti-vWF staining of blood vessels in PBMC+ES Matrigel plug. (F) High power of the anti-podoplanin staining in an IL-6-containing Matrigel plug at 9 days. The scale bars represent 50 microns in A, B, D and E; 100 microns in 5C; and 10 microns in 5F.

The Matrigel plugs were first examined histologically with H&E staining to identify and quantify the cellular infiltration into the central area of the plugs. Different degrees of cellular infiltration were seen in the specific quantification sites of the plugs in different test groups (Fig. 5). The principle cellular elements present were vascular; other cellular elements such as lymphocytes and monocytes were only seen within these vascular elements and not independently in the extra-vascular areas. The presentation of the vascular elements varied from tubular formations (Fig. 5B and 5C) to distinct elongated vessels (Fig. 5D). The number of cells present in the examined areas of the Matrigel plugs varied between the groups, although there was consistency in form and amount within each treatment group. Immunohistochemical staining for the presence of vWF and podoplanin was carried out to identify blood and lymphatic vessels, respectively (Fig. 5E and 5F).

Overall, staining against podoplanin which identifies the lymphatic endothelium was more prevalent in the Matrigel plugs from all groups when compared to anti-vWF staining which identifies the blood vascular endothelium. When comparing different treatments for the presence of lymphatic endothelial elements, Groups 1 (Matrigel alone) and 2 (Unstimulated PBMCs alone) were not significantly different, whereas Groups 3–6, or those containing the ES-stimulated supernatants and lymphangiogenic mediators, had significantly more lymphatic vascular elements than either Group 1 or 2 (Table 2). Plugs from Groups 3–6 had significantly more blood vascular elements than either the control Matrigel alone (Group 1) or unstimulated PBMC Matrigel (Group 2). Assessment of the color intensity by pixel enumeration with either podoplanin or vWF also showed similar significant differences between the groups VEGF-A, IL-8 and IL-6 compared to control samples and a significant difference between the ES-PBMC group compared to the unstimulated PBMC supernatant group (Table S1).

**Table 2:** Quantitative assessment of the presence of podoplanin positive areas (lymphatic endothelial elements) and vWF positive areas (blood endothelial elements) in treated Matrigel plugs recovered from rats 9 days after sub-cutaneous implantation.

	GROUP	PODOPLANIN POSITIVE AREAS (CPA+/− SE)	vWF POSITIVE AREAS (CPA+/− SE)
**1**	MATRIGEL ALONE	0.21 (0.1)	0.18 (0.1)
**2**	UNSTIMULATED PBMCs	2.71 (0.8)	0.08 (0)
**3**	ES-STIMULATED PBMCs	10.50 (2.3)*	3.10 (1.0)***
**4**	IL-6	11.57 (3.9)*	5.10 (1.3)***
**5**	IL-8	5.29 (1.7)**	1.23 (0.3)****
**6**	VEGF-A	12.21 (3.1)*	4.64 (1.2)***

A total of 9 areas were examined in each sample (54 areas per treatment) and assessed using a Chalkley Point Array count (CPA).

* Significantly different (p<0.005) from anti-podoplanin Groups 1 and 2.

** Significantly different (p<0.05) from anti-podoplanin Groups 1 and 2.

*** Significantly different (p<0.005) from anti-vWF Groups 1 and 2.

**** Significantly different (p<0.05 from anti-vWF Groups 1 and 2.

## Discussion

Lymphangiectasia, or the dilation of LVs, and lymphangiogenesis are subclinical features of filarial infection. LVs containing adult worms from infected individuals are characterized as distended, dilated, tortuous and highly indented [40]–[42]. In dilated lymphatics, flow is impaired leading to improper drainage of interstitial fluids. The progression of mild lymphangiectasia to clinical lymphedema may be due to the accumulation of lymphatic fluid in the tissues over time following damage to the LVs. Lymphangiectasia is not restricted to the site of the worm nest, but is found along the length of the infected vessel [8] arguing that a soluble factor secreted by the worm, that can travel the length of the vessel, is responsible for the altered lymphatic pathology. Additionally, lymphangiectasia is greatest near the worm nest and the removal or killing of worms can reduce lymphatic dilation [14]–[16], [40] suggesting living adult worms and their ES products have the strongest biological effects locally and are associated with altering lymphatic pathology.

A number of factors may play a role in the development of lymphangiectasia and our data suggest that parasite products are central in this process. Since no direct effects of ES products on LECs were detected, we hypothesized that ES products activate the lymphatic endothelium indirectly through an accessory cell [43]. Here, we have demonstrated that *Brugia* ES products stimulate host cells to produce lymphangiogenic mediators such as IL-8, IL-6 and VEGF-A. Autocrine stimulation by these molecules on the PBMCs themselves may have also amplified the response in our system. Next, we demonstrated these same mediators altered LEC phenotypes. Moreover, the mediators tested in this study not only induced LV formation *in vivo* using a Matrigel plug model, but these mediators also induced angiogenesis. Therefore, the production of these molecules could contribute to the development of lymphangiectasia in filarial-infected individuals.

Other studies have supported the role of parasite molecules in lymphangiogenesis and lymphangiectasia. Bennuru et al. showed microfilariae stimulate LEC proliferation and alter LEC junction adherence pathways which could contribute to lymphatic dilation [44]. Microfilariae may also contribute to the development of lymphatic disease as this stage is released simultaneously with adult ES products and microfilarial ES is found in our adult worm ES. Others have proposed that parasite endosymbiont *Wolbachia* is responsible for elevated lymphangiogenic mediators, but Bennuru et al. elegantly demonstrated that the levels of VEGF-A, VEGF-C and VEGF-D pre- and post-DEC treatment did not change suggesting a minimal role for *Wolbachia* [27]. Bacterial infection, including *Wolbachia*, has been linked with IL-8 production [45], so the levels of other lymphangiogenic mediators such as IL-8 and IL-6 will also need to be examined in this setting. Furthermore, human ECs exposed to live intact microfilariae either carrying or free of *Wolbachia* or not, only induced a limited number of cytokines and angiogenic mediators suggesting *Wolbachia* is not a strong stimuli altering the EC phenotype [46].

In this present study, we aimed to mimic the relationship between the living adult worm and the lymphatic endothelium, and not the changes associated with dead worms, thus we used *Brugia* ES products rather than adult worm or microfilariae extracts. Crude extracts would be more representative of stimuli associated with worm death, a different scenario. Upon worm death, there is an immense inflammatory reaction which is distinct from the lack of inflammation associated with the presence of the living worm. Responses to living worms differ histopathologically from the granulomatous responses seen with dead worms (Mackenzie, unpublished observations). Monocytes/macrophages appear to be central in both responses, although they may be acting differently in each situation. Filarial ES products are generally thought to be immunosuppressive but here ES induced PBMCs to produce IL-8 and IL-6 which can lead to a massive recruitment of inflammatory cells. However, the lack of inflammation adjacent to living worms suggests IL-8 and IL-6 production does not lead to a massive inflammatory reaction *in vivo*. In contrast, worm death either by drug treatment or natural attrition may exacerbate the development of lymphatic pathology if the acute inflammatory reaction provides a stimulus for downstream processes leading to lymphatic insufficiencies. Future studies will be needed to compare the production of lymphangiogenic mediators and the induction of LVs *in vivo* in response to ES products versus crude extracts.

Even though the expression of lymphangiogenic mediators is generally perceived to be beneficial for the formation of new LVs and to reverse malfunctioning LVs [47]–[49], the over-expression of lymphangiogenic molecules over an extended period of time has been shown to be detrimental and to impair lymphatic function. A massive expansion of the lymphatic network can lead to defective LVs and thus decreased drainage and lymphedema. For example, VEGF-A and VEGF-C over-expression results in structurally and functionally abnormal and dilated lymphatics [50]–[52]. ES-stimulated host cells may compromise lymphatic function by secreting lymphangiogenic factors over many years throughout the duration of worm infection. It is important to note that a worm infection can last five years or more so the kinetics and molecular mechanisms associated with altering lymphatic pathology may differ from those involved in acute infection and may be cumulative over time. The cumulative amounts/effects of these soluble mediators may parallel those observed in over-expression model systems leading to defective lymphatics. For instance, elevated plasma levels of VEGF-C have been found in microfilaremic individuals compared to endemic normal individuals [28] suggesting the same VEGF and cytokine molecules involved in lymphangiogenesis and lymphangiectasia in other models are also present in filarial infection. These lymphangiogenic cytokines and growth factors may be binding their receptors which are expressed on LECs lining the vessel [20], [53], [54]. Besides the chronicity of filarial infections, worm infections, and specifically worm ES products, are also associated with a down regulation of the immune response so future experiments will also need to address how a chronic infection alters the formation of LVs in the presence of a dampened proinflammatory response.

Even though we did see the production of VEGF-A by PBMCs in response to worm ES, we did not see the production of VEGF-C that was previously shown to be elevated in filarial-infected individuals [28], [31]. We also did not detect elevated levels of VEGF-D or lymphangiogenic cytokines IL-3 or IL-7. The lack of detection of VEGF-C, VEGF-D, IL-3 or IL-7 may be because we were examining the production of these molecules by PBMCs which may not be the cellular source; these molecules may be produced by a cell found focally at the infection site. VEGF-C and VEGF-D signaling through VEGFR-3 is the primary and most well-characterized mechanism contributing to lymphangiogenesis, but there is also an emerging role for VEGF-A in lymphangiogenesis [52], [55]–[57], so it is possible that this molecule may be playing an important role in filarial-induced lymphatic pathologies. In addition to potential systemic versus local differences in lymphangiogenic mediators, differences between individual responses were also noted. The variability in lymphangiogenic mediators, especially for IL-8, produced by PBMCs basally and after ES stimulation made control experiments injecting supernatants from unstimulated PBMCs of paramount importance. Regardless, supernatants from ES-stimulated PBMCs induced significantly more podoplanin and vWF staining compared to supernatants from unstimulated PBMCs. Furthermore, supernatants from unstimulated PBMCs induced more vessel formation than Matrigel alone confirming the basal production of these mediators and providing an important baseline control beyond Matrigel alone.

Monocytes and macrophages play a major role in supporting lymphangiogenesis. They can produce lymphangiogenic factors such as VEGFs and cytokines which induce LEC proliferation, survival, migration and tubule formation [33], [34]. In this present study monocytes were primarily responsible for the production of IL-8 and VEGF-A in response to ES products; however we did not identify the cell type responsible for the production of IL-6, so future experiments need to identify the source of IL-6.

Monocytes and macrophages may play a role in the lymphatic pathology associated with filarial infection. Typically, LVs from infected individuals are thought to be devoid of an inflammatory response [41]; however, some have noted small lymph thrombi composed of mononuclear cells and multinucleated giant cells within the lumen [42]. Here, we defined CD14^+^ cells as the primary producer of IL-8 and VEGF-A in response to *Brugia* ES products and others have also reported the presence of monocytes/macrophages in regions of lymphangiectasia and lymphangiogenesis in *O. volvulus* infection [30], [31]. In nodules isolated from humans infected with *O. volvulus*, the predominant cell type associated with the worms was the macrophage and many macrophages stained positive for the lymphatic-specific marker LYVE-1 [30]. Additionally, some LYVE-1^+^ macrophages were integrating into the lymphatic endothelium [31]. Taken together, these data suggest that monocytes/macrophages are important in lymphangiectasia and lymphangiogenesis in filarial infections and future research is needed to define the role of these cells in lymphatic filariasis.

One could speculate that the worm induces lymphangiogenesis and lymphangiectasia for many reasons. The worm may increase vessel diameter to provide a larger space for habitation; increasing the vessel diameter also slows lymphatic flow and increases the availability of nutrients and resources. The worm may stimulate expansion of the lymphatic network by inducing host production of VEGFs and cytokines to increase LEC proliferation and differentiation as a mechanism of LV dilation. We also demonstrated tubule formation in response to ES-stimulated mediators. Filarial worms may induce the formation of new LVs to expand their biological niche, to maintain flow through a collateral network, or to increase the likelihood that their microfilariae reach the periphery for transmission.

In this study we have begun to dissect the molecular mechanisms involved in the development of lymphangiectasia and lymphangiogenesis; however, similar studies must be carried out in cells isolated from endemic populations to confirm that the same molecules and cell types occur in filarial-infected individuals. Given that parasite products induce the production of lymphangiogenic molecules and that infected persons exhibit lymphangiectasia, we hypothesize that these molecules are elevated in infected individuals. We are currently examining the production of VEGFs and cytokines by microfilaremic individuals, endemic normals and those with lymphedema in response to ES products. Since many infected individuals exhibit lymphangiectasia, which may progress to a lymphedema, we need to define the initial molecular mechanisms responsible for the development of disease. Given many of the lymphangiogenic mediators identified in this study are expressed in a variety of inflammatory settings, we hypothesize that lymphangiogenesis is a hallmark of inflammation. Therefore, understanding the pathogenesis of lymphatic filariasis may identify potential molecular targets for preventing disease initiation and progression as well as a greater understanding of the molecular mechanisms associated with lymphatic pathologies from cancer and inflammation.

## Supporting Information

Table S1 Quantitative assessment: Assessment of the presence of vWF or podoplanin positive elements on vascular structures in the SA by measuring the number of positive pixels in a standard area (4 sq.mm) and the difference is related to the Matrigel alone control. A total of 3 areas (and 3–4 fields) per Matrigel plug were quantified, and there were 6 animals tested per treatment, except there were only 5 animals in Group 2. * p<0.005 statistically significant differences between Group 2 and Group 3 for both immunostains tested.
(DOCX)
