# Supplementary material for: Filarial Excretory-Secretory Products Induce Human Monocytes to Produce Lymphangiogenic Mediators
Source: PLoS Negl Trop Dis. 2014 Jul 10;8(7):e2893. doi: 10.1371/journal.pntd.0002893 (PMC4091784; doi:10.1371/journal.pntd.0002893)
Supplement: Table S1 — Quantitative assessment: Assessment of the presence of vWF or podoplanin positive elements on vascular structures in the SA by measuring the number of positive pixels in a standard area (4 sq.mm) and the difference is related to the Matrigel alone control. A total of 3 areas (and 3–4 fields) per Matrigel plug were quantified, and there were 6 animals tested per treatment, except there were only 5 animals in Group 2. * p<0.005 statistically significant differences between Group 2 and Group 3 for both immunostains tested. (DOCX) [file pntd.0002893.s001.docx]

**Supplemental Table 1.** Quantitative assessment: Assessment of the presence of vWF or podoplanin positive elements on vascular structures in the SA by measuring the number of positive pixels in a standard area (4 sq.mm) and the difference is related to the Matrigel alone control.

| GROUP | |  |  |  |  |
| --- | --- | --- | --- | --- | --- |
|  |  | **vWF POSITIVITY**  **(+/- SD)*** | **DIFFERENCE FROM CONTROL** | **PODOPLANIN POSITIVITY**  **(+/- SD) *** | **DIFFERENCE FROM CONTROL** |
| 1 | MATRIGEL ALONE  (i.e. CONTROL) | 0.56 (0.2) | _ | 1.12 (0.6) | _ |
| 2 | UNSTIMULATED PMBCs | 1.11 (0.4) | 2.0 | 2.23 (1.0) | 2.0 |
| 3 | ES-STIMULATED PMBCs | 2.17 (1.1) | 3.9 | 5.31 (1.9) | 4.7 |
| 4 | IL-6 | 3.94 (1.8) | 7.0 | 5.82 (2.4) | 5.2 |
| 5 | IL-8 | 2.01 (1.1) | 3.6 | 2.43 (2.0) | 2.2 |
| 6 | VEGF-A | 3.45 (1.8) | 6.2 | 6.27 (2.3) | 5.6 |

* There was statistically significant differences between Group 2 and Group 3 (p < 0.005) for both immunostains tested. A total of 3 areas (and 3-4 fields) per Matrigel plug were quantified, and there were 6 animals tested per treatment, except there were only 5 animals in Group 2.
